# A Meta-Analysis of the Association between the CC Chemokine Ligand 5 (CCL5) -403 G>A Gene Polymorphism and Tuberculosis Susceptibility

**DOI:** 10.1371/journal.pone.0072139

**Published:** 2013-08-28

**Authors:** M. Y. Areeshi, Raju K. Mandal, Aditya K. Panda, Shafiul Haque

**Affiliations:** 1 Department of Medical Microbiology, College of Nursing and Allied Health Sciences, Jazan University, Jazan, Saudi Arabia; 2 Department of Urology, Sanjay Gandhi Post Graduate Institute of Medical Sciences, Lucknow, Uttar Pradesh, India; 3 Department of Infectious Disease Biology, Institute of Life Sciences, Bhubaneswar, Odisha, India; 4 Department of Biosciences, Jamia Millia Islamia (A Central University), New Delhi, India; The Ohio State University, United States of America

## Abstract

**Aim:**

Many case-control studies have been performed in the recent past to investigate the association between CCL5 -403 G>A (rs2107538) gene polymorphism and tuberculosis (TB) susceptibility in various ethnic groups. However, these studies have produced inconsistent and contradictory results. In the present study, meta-analysis was performed to assess the association between CCL5 -403 G>A polymorphism and TB risk.

**Methodology:**

Quantitative synthesis was done for the published studies based upon association between CCL5 -403 G>A polymorphism and TB risk from PubMed (Medline), EMBASE web search. Pooled odds ratios (ORs) and 95% confidence intervals (95% CIs) were calculated for allele contrast, homozygous, heterozygous, dominant and recessive genetic models.

**Results:**

A total of six studies comprising 1638 confirmed TB cases and 1519 healthy controls were included in this meta-analysis. Variant A allele (A vs. G: p = 0.035; OR = 1.301, 95% CI = 1.019 to 1.662) and variant homozygous (AA vs. GG; p = 0.001; OR = 1.520, 95% CI = 1.202 to 1.923) carriers were significantly associated with TB susceptibility. Similarly, recessive model (AA vs. GG+GA: p = 0.016; OR = 1.791, 95% CI = 1.117 to 2.873) also indicated increased TB risk. Whereas, heterozygous (GA vs. GG: p = 0.837; OR = 1.028, 95% CI = 0.791 to 1.335) and dominant (AA+GA vs. GG: p = 0.222; OR = 1.188, 95% CI = 0.901 to 1.567) models failed to show increased risk of developing TB.

**Conclusions:**

This meta-analysis suggests that there is a significant association between the CCL5 -403 G>A polymorphism and increased risk of TB. However, larger well-designed epidemiological studies with stratified case control and biological characterization may be helpful to validate this association.

## Introduction

Tuberculosis (TB) is one of the commonest infectious disease, and remains a major public health concern owing to spread epidemically in many parts of the world [1]. The causative agent of TB is *Mycobacterium tuberculosis* (*M. tuberculosis*) and it leads to approximately 1.5 million annual deaths globally [2]. It is expected that nearly one-third of the world’s population is infected with *M. tuberculosis* infection, only 5–15% of people develop active TB disease during their lifetime [3]. This indicates that host genetic differences may contribute to TB infection. It is widely accepted that TB is a polygenic disease and increasing evidences suggested that genetic variants, especially those belong to immune system confer susceptibility to active TB at the individual level [4], [5]. However, the underlying etiological mechanism of TB infection is still unclear. Thus, it is anticipated that the identification of host genetic factors for TB risk would greatly help in the control of this infectious disease. *M. tuberculosis* has the ability to survive within the host phagocytic cells, and the relation between the host and the bacteria may lead to tissue damage characterized by granuloma formation, tissue necrosis along with development of cavities and, lastly, spreading of the disease [6], [7]. Initially, the migration of immune cells, like, activated monocytes/macrophages to the site of granuloma formation is mainly facilitated by adhesion molecules as well as chemokines/cytokines [8]. Genes encoding chemokines and their cognate receptors play a significant role in the inammatory response during TB infection [9].

Chemotactic chemokine (C-C motif) ligand 5 (CCL5) belongs to the family of CC chemokines, and considered as a major chemokine, mostly involved in immunoregulatory and inflammatory activities owing to their ability to recruit, activate and co-stimulate T-cells and monocytes [10], [11]. CCL5 is also known as RANTES (Regulated on Activation, Normal T cell Expressed and Secreted) [10], [11]. In addition to the trafficking activity, CCL5, like other CC chemokines, plays a major role in co-stimulation of T-cell proliferation and activation of the T-cells localized in the inflammatory lesions [12]–[14]. Such findings enlightened the significance of CCL5 in antimycobacterial immunity.

Many functional polymorphisms in the CCL5 gene have been deciphered in past, among them -403 resulting in a G>A (rs2107538) polymorphism have been identified in the promoter region and shown association with altered transcriptional activity and subsequent expression in human cell line [15]. Based upon the understanding regarding the functional significance of this genetic variant, it has been considered as a potential susceptibility factor for TB. Till now, a relatively few number of studies have been carried out in different populations but their results are remaining inconsistent and conflicting rather than conclusive [16]–[21]. Inconsistencies in their results can be attributed in terms of sample size and ethnic diversity, and individual studies may have low power to detect overall effect. In order to address the questions posed by the individual studies in terms of above mentioned shortcomings, meta-analysis responds appropriately. Meta-analysis is a powerful technique for analyzing cumulative data from studies where individual sample sizes are small and hold lower statistical power [22]. Hence, the current meta-analysis aims to provide a precise and comprehensive evaluation of the association between CCL5 -403 G>A gene polymorphism and TB risk by compiling data from published studies.

## Materials and Methods

### Identification and Eligibility of Relevant Studies

A systematic search was carried out through PubMed (Medline), EMBASE web data-bases covering all research articles published with a combination of the following key words: “CCL5 OR RANTES gene (polymorphism OR mutation OR variant) AND tuberculosis or TB (last updated on March 2013). Potentially relevant genetic association studies were evaluated by examining their titles and abstracts, and all published studies matching the selected eligible criteria were retrieved and incorporated in this study.

### Inclusion and Exclusion Criteria

In order to minimize heterogeneity and to facilitate the proper interpretation of our findings, studies included in the present meta-analysis had to meet all the following criteria: a) assessed the association between -403 G>A polymorphism and susceptibility of TB, b) used a case-control design based on unrelated individuals, c) recruited pathologically confirmed TB patients and TB free controls, d) have available genotype frequency in cases and controls, e) and published in the English language. In addition, when the case-control study was included by more than one article using the same case series, selection was done for the study that included the largest number of individuals. The main reasons for exclusion of studies were, a) overlapping data and b) case-only studies, c) review articles, d) genotype frequencies or numbers not reported. The supporting flowchart (Figure S1) of studies selection is available as supporting information; see Figure S1 (PRISMA 2009 Flow Diagram).

### Data Extraction and Quality Assessment

For each research publication, the methodological quality assessment and data extraction were independently abstracted in duplicate by two independent investigators using a standard protocol and data-collection form according to the inclusion criteria listed above to ensure the accuracy of the data. In case of disagreement on any item of the retrieved data, the problem was fully discussed to reach an agreement. Characteristics abstracted from the studies included first author’s name, year of publication of the report, the country of origin, source and number of the cases and the controls, genotype frequencies, and type of the study.

### Statistical Analysis

Pooled odd ratios (ORs) and their corresponding 95% class intervals (CIs) were calculated to examine the relation between CCL5 -403 G>A polymorphism and TB risk. Heterogeneity assumption was measured by the chi-square based Q-test [23]. Statistical significance level (p-value) >0.05 for the Q-test suggested a lack of heterogeneity among the selected studies. Calculation of pooled ORs were performed either by the fixed effects model or by the random-effects model [24], [25]. Moreover, I^2^ statistics was utilized to quantify inter-study variability, and the larger values showed an increasing degree of heterogeneity [26]. The Hardy-Weinberg equilibrium (HWE) in the control group was estimated by the chi-square test. The measurement of Funnel plot asymmetry was done by Egger’s linear regression test which a linear regression method to estimate the funnel plot asymmetry on the natural logarithm scale of the OR. To determine the significance of the intercept the t-test (p-value <0.05 was considered as representation of statistically significant publication bias) was employed [27]. The statistical analysis for the current meta-analysis study was done by the comprehensive meta-analysis (CMA) V2 software (Biostat, USA). A comparative assessment of ‘meta-analysis’ softwares was done by using http://www.meta-analysis.com/pages/comparisons.html for the selection and utilization of CMA V2.

## Results

### Characteristics of Published Studies

A total of twenty four articles were retrieved by literature search from the PubMed (Medline), EMBASE web-based data-bases. All retrieved articles were assessed by reading their titles and abstracts. The full texts for the potentially relevant articles were further evaluated for their inclusion and suitability for this meta-analysis. In addition to the data-base search, the reference lists of the retrieved articles were further screened for other possible potential studies. As mentioned in the materials and methods section, inclusion and exclusion criteria were set and implemented for the selection of pertinent studies. Studies either using CCL5 polymorphism to predict survival in TB patients or considering CCL5 variants as an indicator for response to therapy were excluded. Research studies measuring the levels of CCL5 mRNA or protein expression or review articles were also excluded. Based upon the selection criteria, only case-control or cohort design based studies having frequency of all three genotypes were included in the current meta-analysis. After cautious screening and following the inclusion and exclusion criteria, six eligible original published studies were included in the present meta-analysis (Table 1). A detailed flowchart of the selection process has been shown in Figure S1. Important parameters, like, distribution of genotypes, HWE p-values of the controls and TB risk are tabulated in Table 2.

**Table 1:** Main characteristics of all studies included in the meta-analysis.

First Author	Year	Country of Origin	Study Design	Genotyping Method	Cases	Controls	Source of Genotyping
Mishra et al.	2012	India	PB	ARMS PCR	215	216	Blood
Selvaraj et al.	2011	India	PB	PCR RFLP	212	213	Blood
Ben-Selma et al.	2011	Tunisia	HB	PCR RFLP	168	150	Blood
de Wit et al.	2011	South Africa	PB	ARMS PCR	505	318	Blood
Sanchez-Castañón et al.	2009	Spain	PB	PCR RFLP	76	157	Blood
Chu et al.	2007	China	HB	PCR RFLP	462	465	Blood

HB: Hospital based, PB: Population based.

ARMS PCR: Amplification Refractory Mutation System -Polymerase Chain Reaction.

PCR-RFLP: Restriction Fragment Length Polymorphism analysis of PCR amplified fragments.

**Table 2:** Genotypic distribution of CCL5 -403 G>A gene polymorphism included in the meta-analysis.

Author(s) and yearof publication	Control				Case					Association
	Genotype			Minor allele	Genotype			Minor allele	HWE	Yes/No
	GG	GA	AA	MAF	GG	GA	AA	MAF	p-value	
−403 G>A (rs2107538)										
Mishra et al., 2012	131	71	14	0.22	125	57	33	0.28	0.30	Yes
Selvaraj et al., 2011	91	97	23	0.33	109	82	21	0.29	0.70	No
Ben-Selma et al., 2011	119	30	1	0.10	149	69	5	0.17	0.54	Yes
de Wit et al., 2011	75	153	81	0.50	122	228	143	0.52	0.86	No
Sanchez et al., 2009	116	39	2	0.13	43	24	9	0.27	0.52	Yes
Chu et al., 2007	214	199	52	0.32	196	173	93	0.38	0.57	No

### Publication Bias

In order to examine the publication bias among the included studies for the meta-analysis, Begg’s funnel plot and Egger’s test were performed. The shape of funnel plots was appearing symmetrical and the results of Egger’s test provided the statistical evidence of the funnel plot. The results demonstrated lack of publication bias among all comparison models (Table 3).

**Table 3:** Statistics to test publication bias and heterogeneity in the meta-analysis.

Comparisons	Egger’s regression analysis			Heterogeneity analysis			Model used for meta-analysis
	Intercept	95% Confidence Interval	p-value	Q value	P_heterogeneity_	I^2^ (%)	
A vs. G	3.09	−3.88 to10.06	0.28	21.73	0.001	76.98	Random
AA vs. GG	1.81	−2.66 to 6.29	0.32	18.12	0.003	72.41	Random
GA vs. GG	3.28	−3.55 to 10.21	0.25	12.27	0.031	59.27	Random
AA+GA vs. GG	3.33	−4.40 to 11.07	0.29	16.65	0.008	68.06	Random
AA vs. GG+GA	1.78	−2.10 to 5.67	0.27	16.81	0.005	70.27	Random

### Test of Heterogeneity

Q-test and I^2^ statistics were employed to test the heterogeneity among the selected publications. Heterogeneity was observed in all models, such as allele (A vs. G), homozygous (AA vs. GG), heterozygous (GA vs. GG), dominant (AA+GA vs. GG) and recessive (AA vs. GG+GA) genotype model, which were included for the meta-analysis. Thus, the random effect model was applied to calculate the pooled ORs and 95% CI (Table 3).

### Meta-analysis of CCL5 -403 G>A Polymorphism and TB Susceptibility

**Figure 1:**
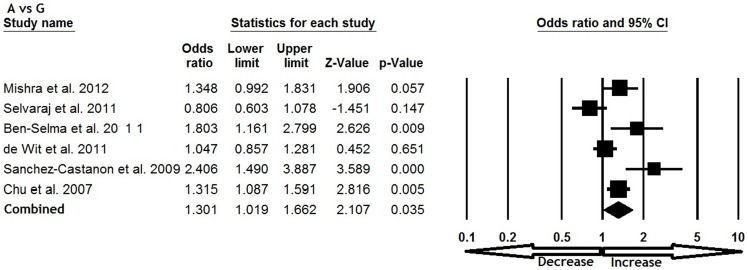
Forest plot and ORs with 95% CI of CCL5 -403G>A polymorphism and TB risk (A vs. G).

**Figure 2:**
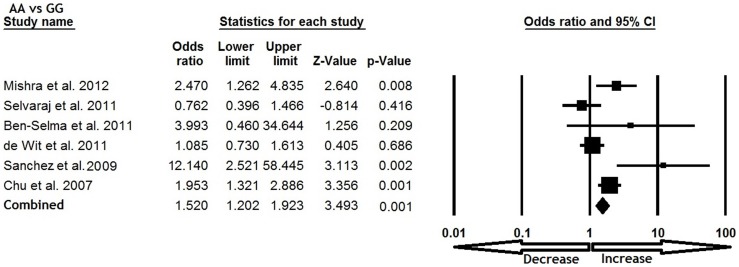
Forest plot and ORs with 95% CI of CCL5 -403G>A polymorphism and TB risk (AA vs. GG).

**Figure 3:**
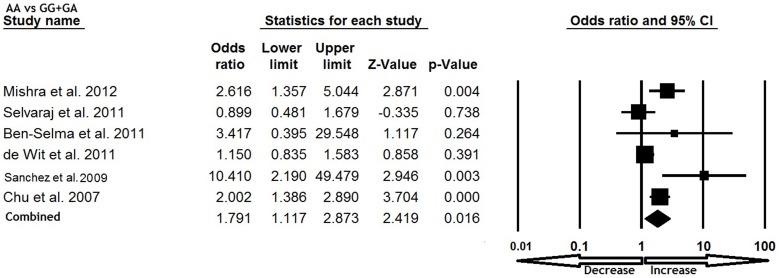
Forest plot and ORs with 95% CI of CCL5 -403G>A polymorphism and TB risk (AA vs. GG+GA).

**Figure 4:**
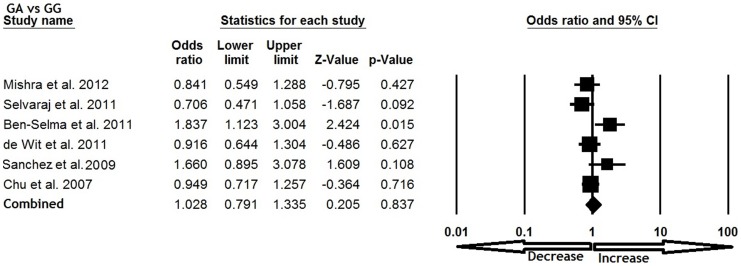
Forest plot and ORs with 95% CI of CCL5 -403G>A polymorphism and TB risk (GA vs. GG).

**Figure 5:**
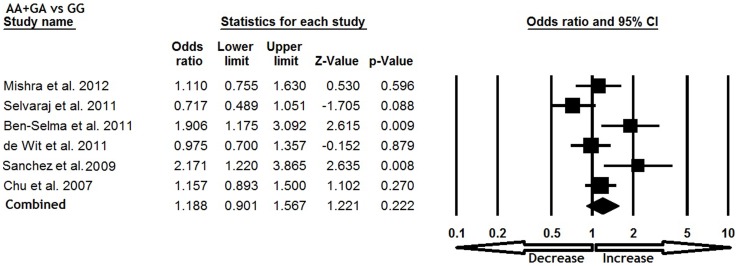
Forest plot and ORs with 95% CI of CCL5 -403G>A polymorphism and TB risk (AA+GA vs. GG).

All six studies were pooled together which resulted into 1638 confirm TB cases and 1519 controls, and the random effects model (based on heterogeneity) was employed to evaluate the overall association between the CCL5 -409 G>A polymorphism and TB susceptibility. The pooled results demonstrated that variant allele A was significantly associated with increased risk of TB (A vs. G: p = 0.035; OR = 1.301, 95% CI = 1.019 to 1.662) (Figure 1). Mutant homozygous genotype AA showed 1.5 fold increased TB risk (AA vs. GG; p = 0.001; OR = 1.520, 95% CI = 1.202 to 1.923) compare with wild type homozygous GG (Figure 2). Furthermore, recessive genetic model also indicated 1.7 fold increased risk of TB (AA vs. GG+GA: p = 0.016; OR = 1.791, 95% CI = 1.117 to 2.873) (Figure 3). However, heterozygous GA genotype (GA vs. GG: p = 0.837; OR = 1.028, 95% CI = 0.791 to 1.335) (Figure 4) and dominant (AA+GA vs. GG: p = 0.222; OR = 1.188, 95% CI = 0.901 to 1.567) model failed to show any altered risk for TB occurrence (Figure 5).

## Discussion

Despite recent advancements in TB diagnosis and antimycobacterial therapy, the prognosis of TB patients remains miserable. It is well established that risk of TB is determined not only by the infection causing agent and environmental factors but also by the host genetic factors [28]. As a result, numerous candidate genes were investigated to evaluate the probable association between modulations of TB susceptibility across different populations. Many studies have been carried out to address the association between CCL5 -403 G>A gene polymorphism and the risk of TB, but all produced varying and contradictory results. Due to the above mentioned conflicting results from relatively individual and small studies underpowered to detect the absolute effects. To improve statistical power and appraise the association of CCL5 -403 G>A polymorphism, we performed a meta-analysis with the collected data to find a more definitive conclusion.

To the best our knowledge, this is the first meta-analysis study addressing the association between CCL5 -403 G>A gene polymorphism and susceptibility of TB, and in this study, a total of six studies were included for the analysis. The pooled results demonstrated that CCL5 -403 G>A polymorphism have substantial effect on the occurrence of TB. Subjects with variant (A) allele and variant homozygous (AA) showed 1.3 and 1.5 fold increased risk of developing TB in comparison with the wild type G allele and homozygous AA genotype. Similarly, recessive model has shown increased risk of TB. CCL5 plays a key role in the antimycobacterial immune response by recruiting mononuclear cells to the site of infection [8], study has shown that the -403 A CCL5 allele is associated with lower serum level of CCL5 [29]. On the basis of above, it can be speculated that -403 G>A polymorphism might be associated with altered (either above or below) concentrations and production of CCL5, this varying range of concentrations may be related with impaired function of this important chemokine and thus increased risk of TB. Knowing the vital role of CCL5 in TB pathogenesis, it is biologically plausible that -403 G>A polymorphism could be a genetic factor for inter-individual differences in susceptibility to TB.

Lastly, the genetic control of the immune response against *M. tuberculosis* infection seems to be polygenic [30], single genetic variant is usually insufficient to forecast the risk of TB. One important property of this gene polymorphism is that their frequency can vary substantially between different races or ethnicities. Despite the significant efforts to test the possible association between CCL5 -403 G>A polymorphism and TB risk in the current meta-analysis, several limitations were there which might have affected the result and must be addressed in future studies. First, we only included studies published in the English language. Second, articles indexed by the selected electronic databases were included; it might be possible that some relevant articles published in other language or indexed in other databases, which may be missed in this study. Third, although we failed to detect any publication bias, but selection bias may exist because only studies published in the English were included. Fourth, in this meta-analysis we found inter-study heterogeneity. Numerous factors might play role to this heterogeneity; ethnicity is one such factor, as allele and genotype allocations for CCL5 -403 G>A locus varied between different ethnic populations, and environmental contacts in various case-control studies were not investigated, these may also affect genetic susceptibility. Fifth, the selected data was not stratified by other factors, like, TB severity and HIV status, and our current findings are based on unadjusted assessment, which limit the estimation of the effects of the gene-environment and gene-gene interactions during TB infection.

## Conclusions

We conclude that meta-analysis is an extremely valuable and economical method which pools both statistically significant and non-significant results from individual similar studies and produces an absolute conclusion [27]. This meta-analysis evaluated the relationship between CCL5 -403 G>A polymorphism and TB risk and suggested that -403 G>A polymorphism appeared to be associated with TB susceptibility. Hence, our meta-analysis results suggest that -403 G>A polymorphism in the chemokine (C-C motif) ligand 5 could be employed as new risk factor for TB and the screening utility of this genetic variant in asymptomatic individuals may be warranted. Though, future well designed studies with larger sample size might be helpful to authenticate this association in different populations including consideration of environmental factors responsible for TB risk. Such studies might eventually lead to a superior and comprehensive understanding of the association between the CCL5 -403 G>A polymorphism and TB risk.

## Supporting Information

Figure S1 **PRISMA 2009 Flow Diagram.**
(TIF)

Checklist S1 **PRISMA 2009 Checklist.**
(DOC)
