# Supplementary figures and images for: A Meta-Analysis of the Association between the CC Chemokine Ligand 5 (CCL5) -403 G>A Gene Polymorphism and Tuberculosis Susceptibility
Source: PLoS One. 2013 Aug 28;8(8):e72139. doi: 10.1371/journal.pone.0072139 (PMC3756059; doi:10.1371/journal.pone.0072139)

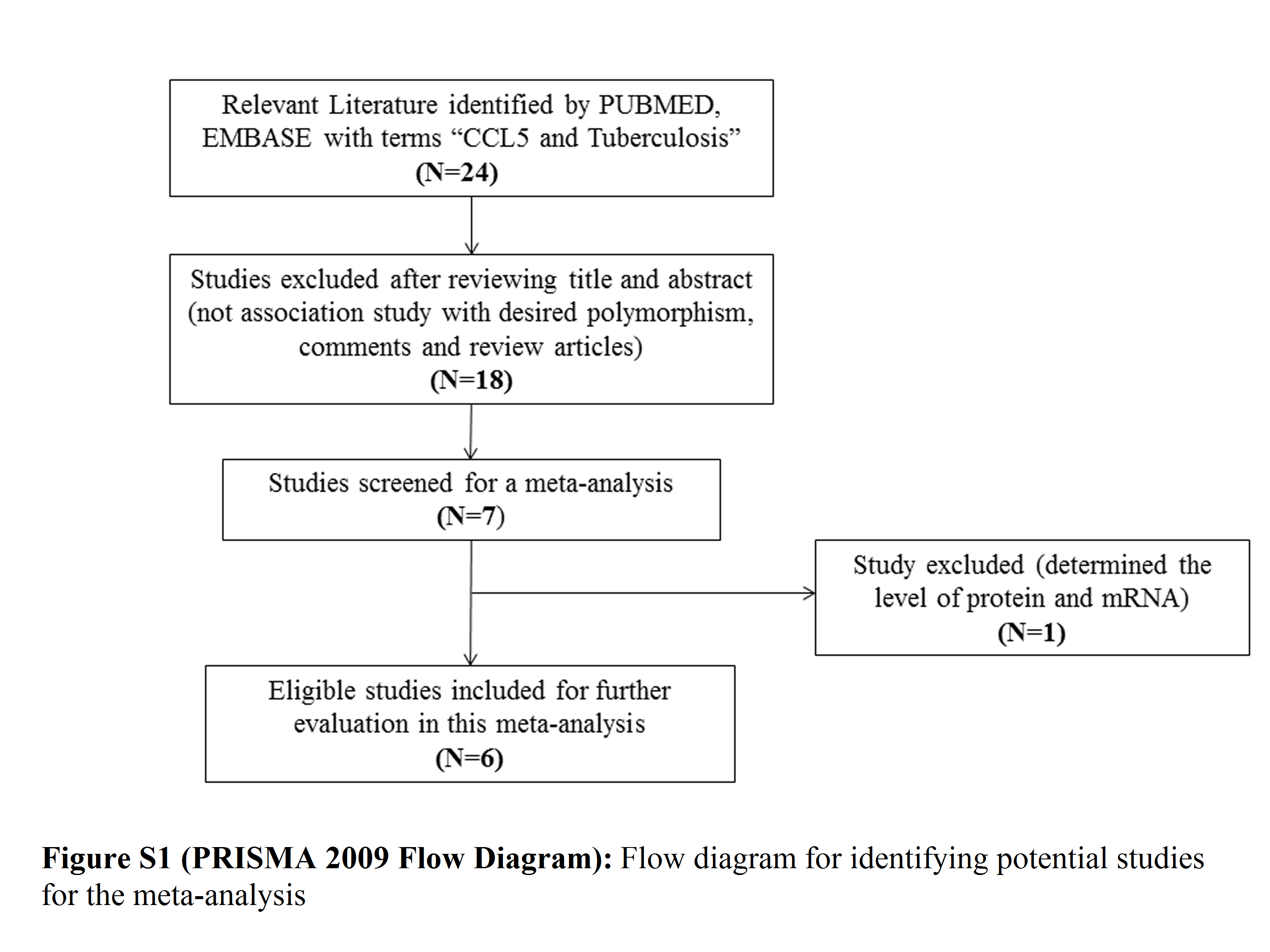

Supplement: Figure S1 — PRISMA 2009 Flow Diagram. (TIF) [file pone.0072139.s001.tif]
